# Adaptations in hepatic glucose metabolism after chronic social defeat stress in mice

**DOI:** 10.1038/s41598-024-76310-3

**Published:** 2024-10-26

**Authors:** Fabiënne S. Meijboom, Annika Hasch, Inigo Ruiz de Azua, Camila Takeno Cologna, Shauni Loopmans, Beat Lutz, Marianne B. Müller, Bart Ghesquière, Michael A. van der Kooij

**Affiliations:** 1 Present Address: Department for Developmental Origins of Disease (DDOD), Wilhelmina Children’s Hospital, University Medical Center Utrecht, Utrecht University, Utrecht, The Netherlands; 2 Leibniz Institute for Resilience Research (LIR), Mainz, Germany; 3 Institute of Physiological Chemistry, University Medical Center of the Johannes Gutenberg University Mainz, Mainz, Germany; 4 Laboratory of Applied Mass Spectrometry, Department of Cellular and Molecular Medicine, KU Leuven, Leuven, Belgium; 5 Center for Cancer Biology, Metabolomics Core Leuven, VIB, Leuven, Belgium; 6 Department of Psychiatry and Psychotherapy, Translational Psychiatry, University Medical Center of the Johannes Gutenberg University Mainz, Mainz, Germany

**Keywords:** Chronic social defeat, Stress, Glucagon, Glucose, Liver, Mass spectrometry, Mice, Homeostasis, Metabolomics, Stress and resilience

## Abstract

Chronic stress has been shown to induce hyperglycemia in both peripheral blood and the brain, yet the detailed mechanisms of glucose metabolism under stress remain unclear. Utilizing ^13^C_6_-labeled glucose to trace metabolic pathways, our study investigated the impact of stress by chronic social defeat (CSD) on glucose metabolites in the liver and brain one week post-stress. We observed a reduction in ^13^C_6_-enrichment of glucose metabolites in the liver, contrasting with unchanged levels in the brain. Notably, hepatic glycogen levels were reduced while lactate concentrations were elevated, suggesting lactate as an alternative energy source during stress. Long-term effects were also examined, revealing normalized blood glucose levels and restored glycogen stores in the liver three weeks post-CSD, despite sustained increases in food intake. This normalization is hypothesized to result from diminished glucagon levels leading to reduced glycogen phosphorylase activity. Our findings highlight a temporal shift in glucose metabolism, with hyperglycemia and glycogen depletion in the liver early after CSD, followed by a later phase of metabolic stabilization. These results underscore the liver’s critical role in adapting to CSD and provide insights into the metabolic adjustments that maintain glucose homeostasis under prolonged stress conditions.

**Supplementary Information:**

The online version contains supplementary material available at 10.1038/s41598-024-76310-3.

## Introduction

Stress-induced imbalances in glucose metabolism are central to stress-related pathophysiology^1,2^. Yet, how glucose metabolism is affected in response to stress remains largely unknown. Interestingly, alterations in glucose metabolism have been documented in major depression^3^, a mental disorder intimately connected to chronic stress. Peripheral hyperglycemia was seen in patients diagnosed with major depression^4^, in conjunction with a decreased cerebral glucose uptake, as measured by ^18^F-FDG uptake^5,6^.

These findings have been replicated in animal models of chronic stress, modeling depression-like phenotypes in rodents. For instance, in mice exposed to chronic social defeat (CSD), we observed peripheral hyperglycemia, which emerged 48 h after stress and lasted for about two weeks^7^. While peripheral- and central glucose levels were increased upon social defeat, cerebral glucose uptake was reduced^7–9^, opening up the possibility that stress-induced hyperglycemia may be unwanted for the brain. In line, normalizing stress-induced hyperglycemia, achieved by providing the animals with anti-diabetic medication (empagliflozine) in their food, restored stress-induced spatial memory deficits^7^.

The liver contributes about 80–90% to endogenous glucose production^10,11^ and is essential for glucose homeostasis^12^. Important herein, the liver also contains the greatest reserve of glucose, in the form of glycogen^13^. Seeing that chronic stress promotes hyperglycemia in the blood, the liver is bound to play an important role in regulating glucose levels under stressful conditions. Nonetheless, few attempts have been made to decipher the impact of chronic stress on hepatic glucose metabolism. We previously found that one week following chronic social defeat stress in mice, hepatic glycogen levels were reduced^14^. Although insulin is a major regulator for glucose metabolism, we did not find differences in insulin concentrations or in insulin tolerance^7^, arguing against a major role for insulin in glucose metabolism after chronic social stress. Glucagon is known to increase hepatic glucose output via glycogenolysis, the breakdown of glycogen, particularly under stressful conditions^15^. We therefore suspected that glucagon would play a critical role in CSD-mediated effects in glucose homeostasis.

To comprehensively study glucose metabolism, we made use of ^13^C_6_-glucose tracing^16^. Herewith we were able to trace glucose-derived metabolites in the brain and liver of mice exposed to chronic social defeat. We expected to observe alterations in glucose metabolism in the brain and/or liver (i.e. affected gluconeogenesis or glycolysis, and/or alterations at the levels of the TCA cycle) of stress-exposed mice that could be instrumental in explaining peripheral hyperglycemia. We then followed up by targeted measurements on enzyme activities and investigated the potential role of glucagon for stress-induced alteration in glucose metabolism.

## Methods

### Animals

Adult male C57Bl/6J mice (‘C57’, Janvier, France) arrived at the animal facility at 8 weeks of age and were habituated to housing conditions (T: 22 ± 2 °C, humidity 50 ± 5%) for at least 1 week before experiments commenced. C57 mice (*n* = 170 in total) were single-housed with food/water *ad libitum* in a 12 h/12 h light-dark cycle. C57 mice were assigned to CSD or non-stressed CTRL at random. Adult male RjOrl: SWISS (CD-1) retired breeders (Janvier, France) were prescreened for aggression and subsequently used as aggressors in the CSD paradigm. Mice were terminated by i.p. injection of Pentobarbital (150 mg/kg b.w.), except for the experiments involving ^13^C_6_-glucose tracing; here mice were decapitated to prevent the impact of the anesthetic on glucose metabolism. Experiments were performed in accordance with the European Directive 2010/63/EU for animal experiments and approved by the local authorities (license N° G20-17-058, Animal Protection Committee of the State Government, Landesuntersuchungsamt Rheinland-Pfalz, Koblenz, Germany) and in compliance with the ARRIVE guidelines. Researchers were blinded while conducting experiments and during analyses.

### Chronic social defeat

The CSD paradigm was conducted for 10 days, as published previously^17^. Briefly, on each day, male C57 mice were sequentially exposed to three social defeats (physical attack) lasting 10 s each in the home cage of three different, unknown, male CD-1 aggressor mice. In between the aggressive encounters, animals were separated for 15 min by a metal grid partition to avoid further physical contact but allowing sensory contact. Following the three daily social defeats, intruder and aggressor mice were housed in the same cage but were physically separated by the grid partition until the next day. CTRLs were exposed to a novel clean cage (90 s) for 10 consecutive days and their home cages were also equipped with a grid partition to mimic the conditions for stressed animals. Following CSD, mice were single-housed for further experimentation.

### CD-1 encounter test

C57 mice were placed in an open field (45 × 45 × 41 cm) with a Plexiglas perforated cylinder (ø 10 cm, H: 20 cm) placed in the center area of the arena containing an unknown male CD-1 mouse. C57 mice were allowed to explore for 5 min and social exploration time was used to calculate the social interaction (SI) index and validate the impact of our social stress paradigm.

### Peripheral measurements for glucose and glucagon

Animals were fasted for 1 h before peripheral blood glucose measurements to exclude variability owing to recent food intake. Peripheral blood was obtained by tail-cut under unrestrained and stress-free conditions^18^; the first drop of blood was always discarded. Morning blood glucose was measured using an electronic handheld glucometer (Accu-Chek; Roche). Three consecutive measurements of blood glucose were taken and averaged to establish reliable values. Glucagon was measured from blood plasma using the manufacturer’s instruction on the Glucagon ELISA kit (AL-157, Ansh Labs, Webster, TX, USA).

### ^13^C_6_-glucose tracing

One week following chronic social defeat, C57 mice were injected (i.p. 2 g/kg b.w.) with ^13^C_6_-glucose (which served as a control for specificity) or with ^13^C_6_-glucose dissolved in saline and we removed the food. Mice were terminated 1.5 h after injection by decapitation. Blood plasma was collected and we extracted the brain and liver. Seeing that the striatum displays high metabolic activity, the striatum was micro-dissected and used to assess glucose metabolism of the brain. Tissue samples were flash-frozen and stored at -80 °C upon metabolite extraction. Samples were homogenized in 80% methanol containing internal standards using a bead homogenizer. After overnight storage at -80 °C, samples were centrifuged (17,000 g for 15 min at 4 °C) and supernatant was analyzed using liquid chromatography-mass spectrometry (MS). Briefly, 10 µl of each sample was loaded into a Dionex Ultimate 3000 LC System (Thermo Scientific Bremen, Germany) equipped with a C-18 column (Acquity UPLC-HSS T3 1. 8 μm; 2.1 × 150 mm, Waters) coupled to a QExactive Orbitrap mass spectrometer (Thermo Scientific) operating in negative ion mode and in full scan mode (m/z range: [70-1050]). Data collection was performed using the Xcalibur software (Thermo Scientific). Data analysis was performed by integrating the peak areas using El-Maven (Elucidata) and subsequent processing using in-house developed software (VIB Metabolomics Core Leuven). Fractional contribution of ^13^C_6_-glucose was calculated based on the following equation:

$$\:total\:contribution\:of\:carbon=\:\frac{{\sum\:}_{i=0}^{n}i*{m}_{i}}{n*{\sum\:}_{i=0}^{n}{m}_{i}}$$

where *n* is the number of carbon atoms in the metabolite of interest, *i* represents the different mass isotopomers, and *m* refers to the abundance of a certain mass.

### Hepatic measurements for glycogen, glycogen phosphorylase, lactate and lactate dehydrogenase

Liver tissue was dissected and stored at -80 °C until processing. Liver tissue was homogenized and processed according to manufacturer’s instructions for glycogen (Glycogen Assay Kit II, colorimetric, ab 169558, Abcam), glycogen phosphorylase (ab27371, Abcam), lactate (L-lactate assay kit, colorimetric, ab65331, Abcam) and lactate dehydrogenase (LDH assay kit, colorimetric, ab102526, Abcam).

### Indirect calorimetry using metabolic cages

Energy expenditure was assessed as described by measuring oxygen consumption (VO_2_) and carbon dioxide production (VCO_2_) by indirect calorimetry^19^. From day 4–7 after CSD paradigm, non-stressed and CSD stressed C57BL/6J mice were individually housed in metabolic chambers (TSE systems GmbH), in which water and food intake, locomotor activity and gas exchanges were monitored. Following 48 h of acclimation (day 4–5 post-CSD), O_2_ consumption, CO_2_ production, locomotor activity, and food and water intake were measured every 15 min for 48 h (day 6–7 post-CSD). Mice had *ad libitum* access to food and water throughout the study.

### Statistical analysis

Values are visualized by individual data points and expressed as mean + SEM. Statistical outliers were defined as values ± twice the S.D. from the mean and were excluded from further analysis. Unpaired two-tailed Student’s *t* tests were used to compare sets of data obtained from two independent groups of animals. To facilitate interpretation of the ^13^C_6_-glucose metabolite data, values are expressed as a Z-score (number of SD away from CTRL). Statistical significance was assessed using a Z-test, with significance Z-scores at ± 1.96 (*P* = 0.05). Data obtained from the metabolic cages and the longitudinal food-intake measurements are tested using a two-way ANOVA with repeated measures. *P*-values are reported in the figure legends, statistical significance was considered at the *P* < 0.05 level. All data were analyzed using Prism version 10 (Graphpad Software Inc.).

## Results

### Stress affects glucose flux in the liver, but not in the brain

**Fig. 1:**
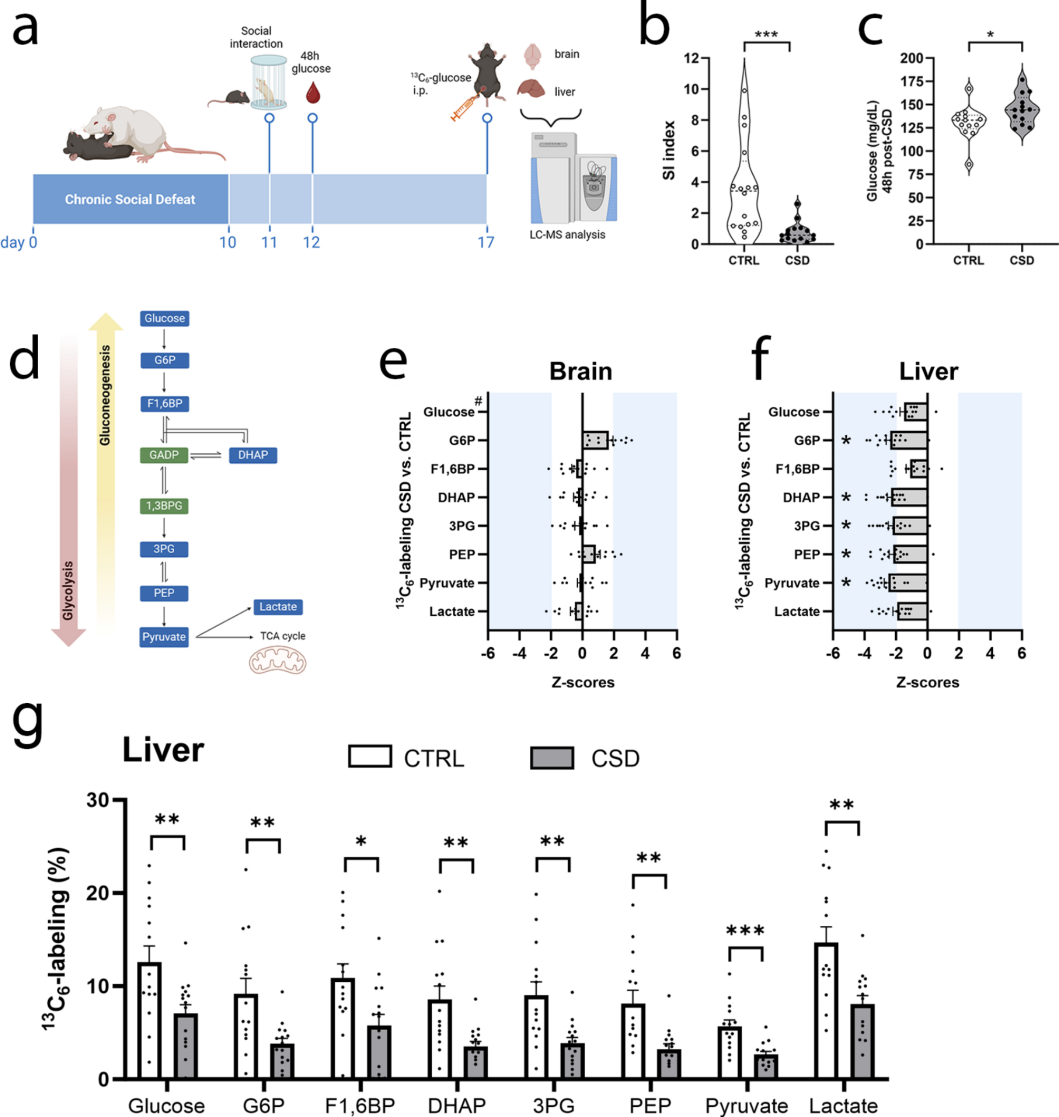
^13^C_6_-glucose tracing reveals altered glucose metabolism in the liver, but not in the brain of stress-exposed mice. (**a**) Schematic representation of the study design. Mice were subjected to chronic social defeat for 10 consecutive days or treated as CTRL. Then, mice were assessed in the social interaction test (day 11), blood glucose was measured (day 12) and we injected mice with ^13^C_6_-glucose after which we extracted the brain and liver (day 17) to be processed for LC-MS analysis. (**b**) CSD-exposed mice did not explore the cylinder containing the unknown CD-1 mouse as much as CTRL (U = 24, *p* < 0.0001, *n* = 16 for CTRL and *n* = 15 for CSD). (**c**) 48 h after CSD, peripheral blood glucose concentrations were increased (U = 42, *p* = 0.028, *n* = 13/group). (**d**) Schematic image displaying metabolites involved in glycolysis/gluconeogenesis ^13^C_6_-labeling was measured in the compounds depicted in blue. We also measured ^13^C_6_-labeling for metabolites of the TCA cycle (Supplementary Figure S2 [Z-scores] and Supplementary Figures S3-4 [% ^13^C_6_-labeling]) ^13^. C_6_-labeling is expressed as a Z-score (number of standard deviations difference from CTRL), to enhance visibility. Z-scores ± 1.96 (blue shaded area) represent significant differences from CTRL. (**e**) ^13^C_6_-labeling was not altered in striatal brain tissue from stressed mice (Z-scores for ^13^C_6_-labeling in G6P: 1.68, *P* = 0.09; F1,6BP: -0.40, *P* = 0.69; DHAP: -0.29, *P* = 0.77; 3PG: -0.21, *P* = 0.83; PEP: 0.85, *P* = 0.40; Pyruvate: -0.05, *P* = 0.96; Lactate: -0.49, *P* = 0.62, *n* = 15/group), # ^13^C_6_-labeled glucose was not detected in the brain tissue. (**f**) We found reduced ^13^C_6_-labeling to be reduced for glucose metabolites of the liver (Z-scores for ^13^C_6_-labeling in glucose: -1.48, *P* = 0.14; G6P: -2.37, *P* = 0.018; F1,6BP: -1.10, *P* = 0.27; DHAP: -2.30, *P* = 0.021; 3PG: -2.22, *P* = 0.026; PEP: -2.18, *P* = 0.03; Pyruvate: -2.49, *P* = 0.013; Lactate: -1.93, *P* = 0.054, *n* = 15/group). (**g**) Here we also display the non-transformed hepatic ^13^C_6_-glucose labeling (%) values for CTRL and CSD: glucose (t = 2.85, df = 27, *P* = 0.008); G6P (t = 3.12, df = 27, *P* = 0.004); F1,6BP (t = 2.66, df = 27, *P* = 0.01); DHAP (t = 3.38, df = 27, *P* = 0.002); 3PG (t = 3.35, df = 27, *P* = 0.002); PEP (t = 3.27, df = 27, *P* = 0.003); pyruvate (t = 4.17, df = 27, *P* = 0.0003) and lactate (t = 3.56, df = 27, *P =* 0.001). Data are presented as individual values in violin plots (**a-b**), Z-scores (**e** + **f**) and as mean + SEM (**a-g**); *P < 0.05; ***P < 0.001. Mann-Whitney tests in **b-c**; Z-tests in **e-f** and Student’s t-test for **g**. G6P: glucose-6-phosphate; F1,6BP: fructose 1,6 biphosphate; DHAP: dihydroxyacetone phosphate; 3PG: 3-phosphoglyceric acid, PEP: phosphoenolpyruvate. Panels **a** and **d** were created with BioRender software.

The day after the chronic social defeat (CSD) paradigm, mice were assessed in the social interaction test and blood glucose levels were taken (Fig. 1a). The week following CSD, mice were injected with ^13^C_6_-glucose and, using LC-MS, glucose metabolism was assessed in brain tissue and liver (Fig. 1a). Stress-exposed mice displayed reduced social interest (Fig. 1b) and increased plasma glucose concentrations 48 h post-CSD (Fig. 1c). Glycolytic intermediates are shown, signifying the directions of glycolysis and gluconeogenesis. LC-MS analyses were performed on the compounds shown in blue (Fig. 1d). Although ^13^C_6_-labeling of glycolytic intermediates was unchanged in the brain (Fig. 1e, Supplementary Figure S1) ^13^, C_6_-labeling of glycolytic intermediates was reduced in the liver (Fig. 1f-g). Mass spectrometry analyses were also performed to study TCA cycle intermediates, where CSD did not affect ^13^C_6_-labeling in either brain (striatal tissue) or liver (Supplementary Figures S2-4). We then hypothesized that at one week post-CSD, we would either find (i) reduced glycolysis (contingent upon increased glycogen levels) or (ii) the involvement of alternative sources for glycolysis in the liver of stressed mice.

### Increased hepatic lactate suggests alternative carbon source for glycolysis in stressed mice

**Fig. 2:**
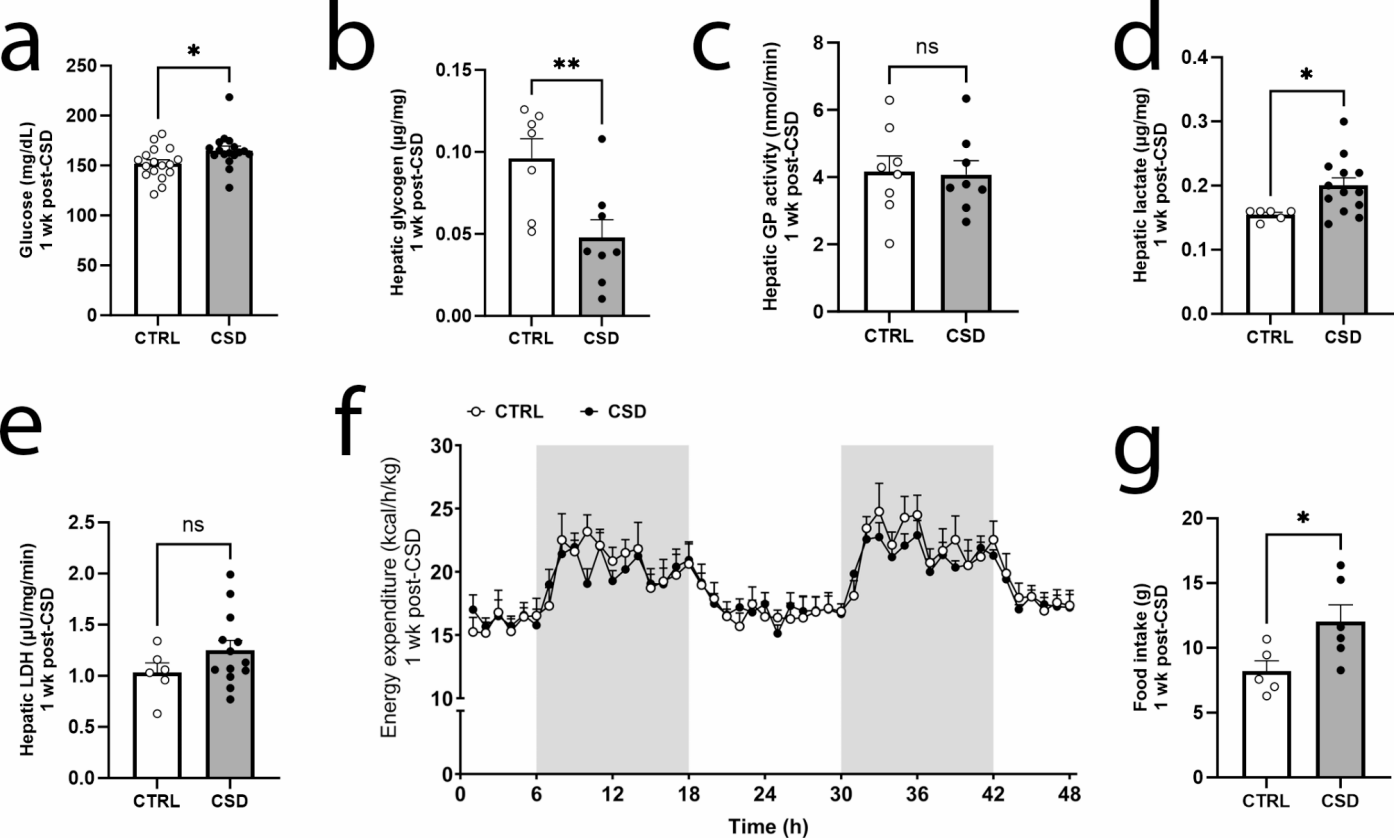
The week following stress, liver alterations include lowered glycogen levels and increased concentrations of lactate. (**a**) Peripheral blood glucose was increased in stressed mice one week post-CSD (t = 2.27, df = 32, *P* = 0.03, *n* = 17/group). (**b**) Hepatic glycogen concentrations were decreased in CSD-exposed mice (t = 3.02, df = 13, *P* = 0.0098, *n* = 7 for CTRL and *n* = 8 for CSD) whereas (**c**) GP activity was not different between CTRL and CSD (t = 0.14, df = 14, *P* = 0.89, *n* = 8/group). (**d**) Lactate levels were elevated in the liver of stressed mice (t = 2.44, df = 17, *P* = 0.026, *n* = 6 and *n* = 13 for CSD), but (**e**) enzyme activities for LDH remained unaltered (t = 1.36, df = 17, *P* = 0.19, *n* = 6 for CTRL and *n* = 13 for CSD). (**f**) We found no evidence for alterations in energy expenditure between CSD and CTRL in metabolic cages (F_1 − 10_= 0.087, *P* = 0.77, *n* = 6/group). The nocturnal phase is indicated by the grey blocks. (**g**) In the metabolic cages, CSD-exposed mice displayed increased food intake (t = 2.40, df = 9, *P* = 0.0396, *n* = 5 for CTRL and *n* = 6 for CSD). Data are presented as mean + SEM; *P < 0.05; **P < 0.01. Student’s t-test in **a-e** and **g**, two-way repeated measures ANOVA in **f**. GP: glycogen phosphorylase, LDH: lactate dehydrogenase.

One week after CSD we still found increased glucose concentrations in the peripheral blood (Fig. 2a), but reduced hepatic glycogen levels (Fig. 2b). However, activity of hepatic glycogen phosphorylase (GP), the rate-limiting enzyme involved in the conversion of glycogen to glucose, was not affected (Fig. 2c). Interestingly, hepatic lactate levels were increased one week post-CSD (Fig. 2d). Taking into account that ^13^C_6_-labeling for lactate was reduced, the increased levels of hepatic lactate suggest there might be an alternative source fueling glycolysis. Hepatic lactate dehydrogenase activity did not differ between CTRL and CSD (Fig. 2e). Therefore, we wondered whether the increased hepatic lactate levels were connected to increased anaerobic metabolism of the animals and tested the mice in metabolic cages. Energy expenditure (Fig. 2f), locomotor activity (Supplementary Figure S5a) and bodyweights (Supplementary Figure S5b) did not differ between CSD and CTRL mice. However, CSD-exposed mice exhibited increased food intake in the metabolic cages as compared to CTRL mice (Fig. 2g). Thus, we presume that increased food intake seen one week post-CSD was linked to the concomitant hyperglycemia.

### The stress-associated decrease in glucagon levels can explain reduced hepatic glycogen phosphorylase activity and may underlie the restored glycogen/glucose balance found three weeks post-CSD

**Fig. 3:**
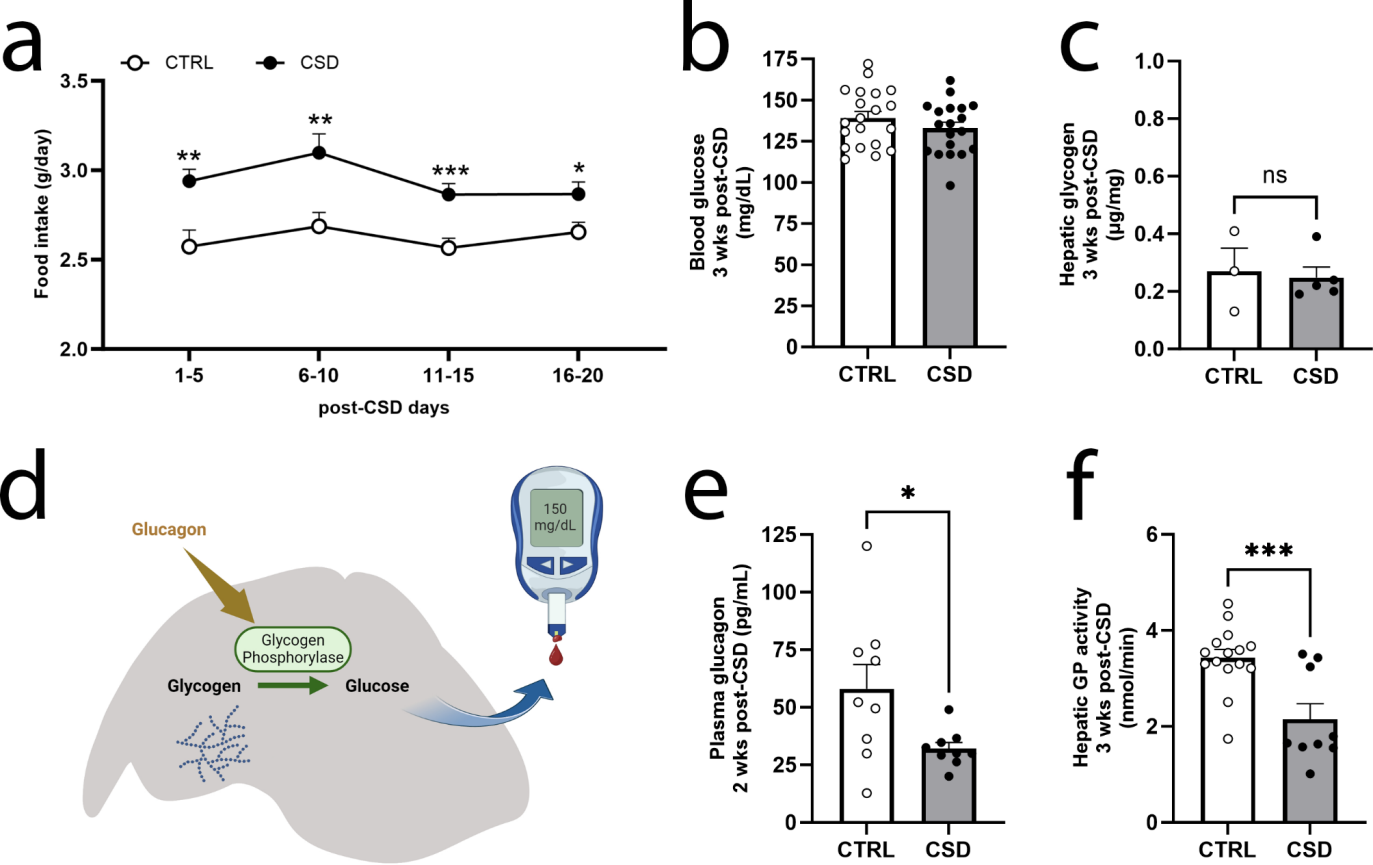
At long term, normalization of blood glucose and liver glycogen levels are associated with a reduction of plasma glucagon and hepatic GP activity. (**a**) Daily food intake remained increased for the three weeks following stress (stress effects: F_1,35_= 14,25, *P* = 0.0006 and post-tests at 1–5 [t = 3.23, df = 31.91, *P* = 0.003], 6–10 [t = 3.13, df = 31.48, *P* = 0.004], 11–15 [t = 3.69, df = 34.25, *P* = 0.0008], 16–20 [t = 2.43, df = 32.96, *P* = 0.021], *n* = 18–19/group). (**b**) Peripheral blood glucose levels from stressed mice and CTRL were similar at three weeks post-CSD (t = 1.19, df = 37, *P* = 0.24, *n* = 19–20/group). (**c**) Hepatic glycogen levels did not differ between CSD and CTRL (t = 0.29, df = 6, *P* = 0.78, *n* = 3 for CTRL and *n* = 5 for CSD). (**d**) Schematic image of the liver signifying the importance of glycogen phosphorylase (GP) and glucagon for the conversion of glycogen into glucose. (**e**) Plasma glucagon levels were reduced for stressed mice, 2 weeks post-CSD (t = 2.39, df = 16, *P* = 0.03, *n* = 9/group) and we also found (**f**) lower GP activity levels for CSD-exposed mice at 3 weeks post-CSD (t = 3.846, df = 22, *P* < 0.001, *n* = 15 for CTRL and *n* = 9 for CSD). Data are presented as individual values and as mean + SEM. *P < 0.05; **P < 0.01; ***P < 0.001. Two-way ANOVA repeated measures with LSD post-test in **a** and Student’s t-tests in **b**, **c**, **e** and **f.** GP: glycogen phosphorylase.

Increased food intake is not only seen one week post-CSD (Fig. 2g), but was also observed thereafter (e.g. three weeks post-CSD) (Fig. 3a). We asked how this overeating affected glucose metabolism and found that at three weeks post-CSD, blood glucose levels are again within the normal range (Fig. 3b), as previously published^7^. Simultaneously, hepatic glycogen levels did not differ between CSD and CTRL (Fig. 3c). Hence, the surplus energy, derived from the hyperphagia at three weeks post-CSD, may serve to restore the depleted glycogen levels, rather than promote glucose into circulation. Additionally, we also observed increased gene transcription levels for phosphoenolpyruvate carboxikinase, pyruvate carboxylase and alanine transferase exclusively at long-term (3 weeks post-CSD), but not at short-term (1 week post-CSD) in stressed mice compared to non-stressed mice (Supplementary Table 1). Glucagon stimulates glucose release into the bloodstream by promoting glycogen phosphorylase (GP) activity, the rate-limiting enzyme to convert glycogen into glucose-1-phosphate (Fig. 3d). However, we find reduced glucagon levels in the sub-acute phase (two weeks post-CSD) (Fig. 3e). As expected, in response to decreased glucagon, enzymatic activity of hepatic GP was reduced as well (Fig. 3f).

### Hypothesized mechanism underlying stress-induced hyperglycemia and its normalization thereafter

**Fig. 4:**
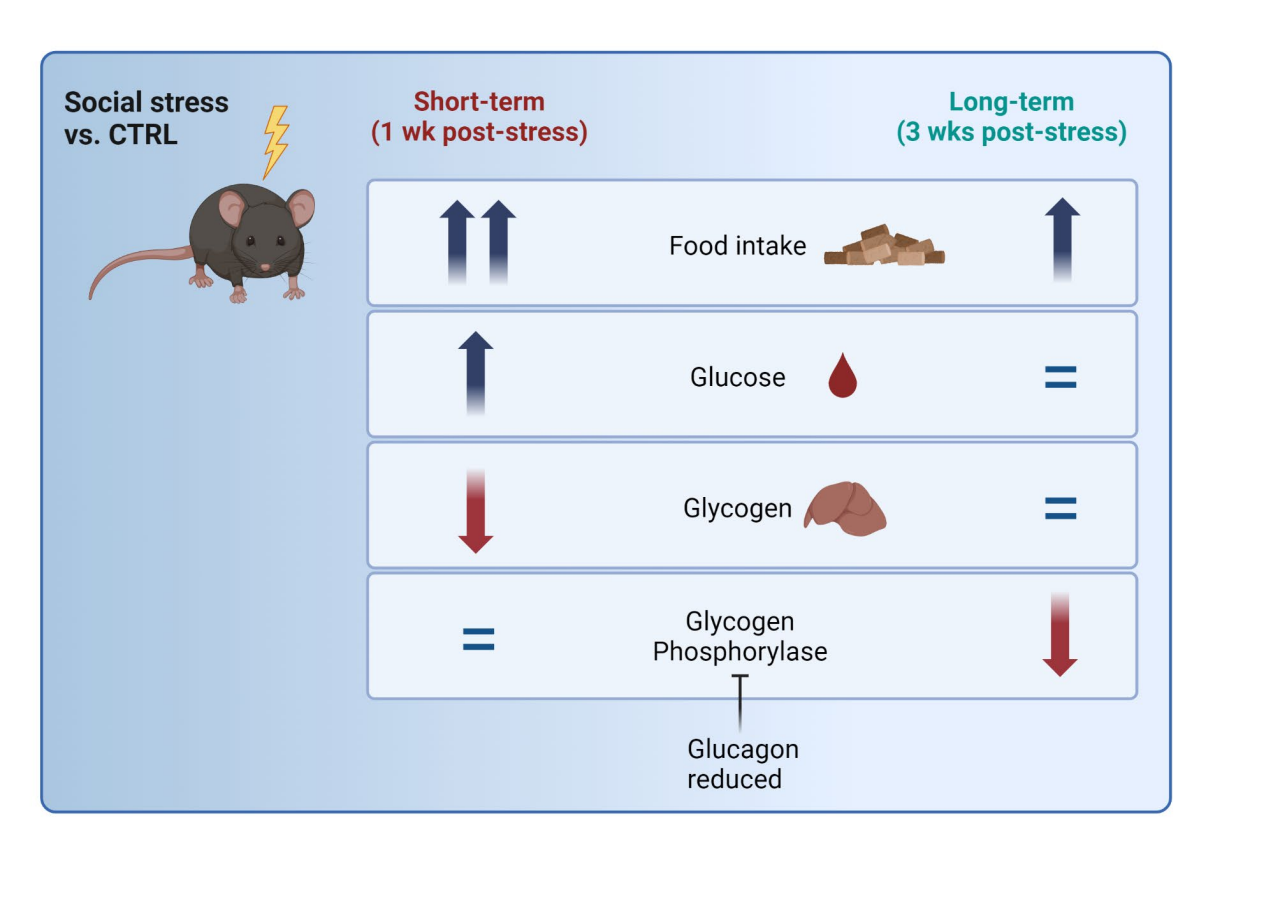
Schematic overview displaying the central findings of this study. We observed metabolic differences between stressed (CSD) and CTRL mice at short-term (1 week following stress) and at long term (3 weeks after stress). At short-term, food intake was strongly enhanced and coincided with peripheral blood hyperglycemia and hepatic glycogen depletion. These effects did not involve changes in the activity of glycogen phosphorylase, the rate limiting step converting glycogen into glucose. At long-term, food intake was still elevated, albeit less intense than at short-term. Both glucose levels in the peripheral blood as well as glycogen concentrations in the liver of CSD-exposed mice did not differ from CTRL mice. However, at long-term we observed a reduction of glycogen phosphorylase activity, which would favor retention of hepatic glycogen at the expense of freely moving glucose into circulation. We suspect that the reduced activity of glycogen phosphorylase was induced by the preceding drop in plasma glucagon levels.

To sum up, at short-term (one week post-CSD), we observed a strong increase in food intake (Fig. 4). We expect that this increased food intake fueled peripheral hyperglycemia and that glycogen conversion to glucose in the liver, evidenced by the depleted hepatic glycogen stores, probably contributed as well. At this timepoint, GP activity was not altered. At long-term (three weeks post-CSD), food intake was increased, although the effect size was more modest and hepatic glycogen levels were at control values. Therefore, we anticipate that the increased food intake at this timepoint served to replete hepatic glycogen stores, rather than sustaining hyperglycemia in the peripheral blood. We hypothesize that the increased hepatic glycogen/blood glucose balance at long-term, comparing the conditions between three and one week post-CSD, was accomplished via a reduction in GP activity, prompted by lowered plasma glucagon levels.

## Discussion

Chronic stress leads to hyperglycemia in the peripheral blood as well as in the brain^3^, but it remains unclear how glucose metabolism is affected in detail. Making use of ^13^C_6_-labeled glucose, as tracer, we investigated ^13^C_6_-enrichment in metabolites from liver and brain, one week after stress. We found lower ^13^C_6_-content for glucose-derived metabolites in the liver, whereas no differences were observed in the brain. Glucose breakdown (glycolysis) may have been decreased in the liver of stressed mice if elevated levels of stored glucose (glycogen) were found. However, hepatic glycogen levels were lower in stressed mice, in line with previous work^14^, thus arguing against CSD affecting glycolysis. We then explored long-term effects of CSD on liver glucose metabolism. We found at three weeks post-CSD that, although food intake remained increased, blood glucose concentrations did not differ between stressed mice and controls. We propose that the diminished plasma glucagon levels observed caused de-stimulation of GP. In turn, a reduced GP activity would explain the absence of hyperglycemia at long term as well as the restored glycogen levels in the liver.

Since the mice used in our study were impacted by the stressor in a similar manner as reported before, the subjects were both suitable and representative to study glucose metabolism. First, social interaction (SI) index for an unknown CD-1 mouse was reduced for stressed animals^20–22^. Second, peripheral blood glucose levels were increased both at 48 h and at one week post-CSD, in agreement with our previous findings^7^.

For our ^13^C_6_-glucose tracing study, we opted to give the mice a peripheral injection, rather than a liquid solution^16^. We deemed it important to precisely control the timing and quantity of the injected tracer, which is less feasible when providing the tracer via the animals’ drinking water. Animals were injected with a dose of 2 g/kg ^13^C_6_-glucose, which corresponds to glucose doses used in the glucose tolerance test (GTT) from previous studies^7,17^. Mice were sacrificed 90 min after injection of the glucose bolus, since we knew from published GTT-results that at this timepoint glucose levels were past their peak, but elevated from baseline. We therefore expected 90 min to be optimal to analyze ^13^C_6_-labeled metabolites. As ^13^C_6_-content differed considerably between glucose-derived metabolites, we decided to express the data not only as raw % of ^13^C_6_-glucose labeling, but also as Z-scores compared to controls, thereby improving visibility. Since previous findings reported a reduction in cerebral glucose uptake, perhaps facilitated by a downregulation of the glucose transporter 1^7,9^, we anticipated that the glucose flux in the stressed brain may have been altered as well. However, the ^13^C_6_-glucose tracing results obtained by LC-MS (Fig. 1) prompted us to focus on the liver, rather than the brain.

In the liver we observed that glycogen was reduced and lactate was increased one week post-CSD. Interestingly, rats exposed to chronic unpredictable mild stress were also found to exhibit decreased liver glycogen content as well as increased lactate concentrations^23^, suggesting common underlying mechanisms related to glucose metabolism, between CSD and chronic unpredictable mild stress^3^. We found an interesting dichotomy between short term (1 week post-CSD) and long term (3 weeks post-CSD) effects of stress on the glucose/glycogen balance. At short term, circulating glucose levels were high and associated with low hepatic glycogen levels but at long term, the balance stabilizes with normalized peripheral glucose levels and repleted glycogen levels in the liver. At first glance, our glycogen findings appear at odds with other research demonstrating that glucocorticoids increase glycogen storage, by stimulating glycogen synthase^24,25^. It needs to be considered, however, that we measured hepatic glycogen levels one week after chronic social defeat; at this time corticosterone levels were not elevated for stress-exposed mice^7^.

The increased levels of lactate found in stressed mice may be congruent with earlier findings on stress hyperlactatemia^26^. The source of lactate during stress, however, may not be so obvious and can be connected to anaerobic glycolysis but could also result from increased aerobic lactate production^26^. Seeing the connection between exercise and lactate^27^, we decided to test stress-exposed mice in metabolic cages. We found no differences in energy expenditure, nor in the amount of locomotor activity. Before- as well as after the metabolic cages we also observed no differences in body weight (Supplementary Fig. 5b), in line with previously published findings, where mouse bodyweights also did not differ between CSD-exposed mice and controls^7,17^. Several mechanisms may underlie the lack of body weight differences between CSD and CTRL including altered thermogenesis^28^, changes in gut microbiota^29^ or increased lipid turnover in stressed mice^30^. Although stressors have been reported to increase energy expenditure in metabolic cages^31,32^, the metabolic status in this work was assessed several hours post-stress, whereas we tested mice one week following CSD. For now, we conclude that one week post-CSD, the increased lactate levels found in the liver are not coupled to alterations in physical activity.

Despite the lack of differences seen in the metabolic cages, these stressed mice retained their increased food intake, as reported before^17^. In another batch of mice, we found that increased food intake was long lasting and still observed three weeks post-CSD. At this time point, peripheral glucose levels were normalized, in line with previous findings^7^. We hypothesize that glucagon is an important mediator in explaining both hyperglycemia at short term and the normalization of blood glucose levels at three weeks after CSD. First, elevated levels of plasma glucagon have been observed after acute stressful stimuli^33,34^. This surge in glucagon then contributes to hepatic glucose output by stimulating glycogenolysis, leading to hyperglycemia^15^. These findings are also consistent with the reduction in hepatic glycogen -suggesting glycogenolysis- and concomitant hyperglycemia, seen one week post-CSD. Thus, in the framework of our CSD-paradigm, we anticipate an approximately one week delay between expected glucagon surges -due to acute stress- and evident hyperglycemia. Similarly, we argued that, if glucagon changes also contribute to a normalization of peripheral glucose concentrations (three weeks after CSD), we would expect this glucose normalization to be preceded by a glucagon-drop one week before. Hence, we measured glucagon at two weeks post-CSD. Mechanistically, glucagon is known to increase GP activity via activation of protein kinase A^35^. Therefore, at long term, the lowered glucagon levels observed for stressed mice may have been responsible for the reduced GP activity in the liver, thereby favoring hepatic glycogen retention over glucose liberation, and leading to a normalization of the hepatic glycogen/blood glucose balance.

We acknowledge limitations to our study. First ^13^C_6_-glucose tracing has only been performed at short term (one week post-CSD) and, although interesting, was not performed at other time points (i.e. at long-term). Second, it was often not feasible to take multiple measurements from the same sets of animals, which sometimes led to uneven number of animals per group and prevented us from including potentially interesting correlational analyses (e.g. correlating glucagon with GP activity). Although beyond the scope of this study, it is important to acknowledge that glycogen plays a larger role than glucose storage alone, and is also relevant for cell differentiation, signaling and redox regulation^36^. Finally, it is common to stratify stress-impacted subjects into susceptible and resilient populations based on the social interaction (SI) index. We decided not to include this approach since we found that our stressed mice uniformly displayed a low SI index, and thus missed the basis for correct stratification. Additionally, we observed that the impact of stress on glucose flux was robust and displayed little variation, therefore limiting the potential benefit to stratify within our CSD experimental group.

To conclude, at short-term, stress-induced adjustments in glucose metabolism take place in the liver, rather than the brain. Of note, these metabolic alterations in the liver may well have been orchestrated by the brain, seeing that hypothalamic control of hepatic glucose production is pivotal in energy homeostasis^37^. At short term, the metabolic changes we observed in the liver appear to favor hyperglycemia at the expense of hepatic glycogen. At long term, the glucose/glycogen balance tips, whereby blood glucose and hepatic glycogen concentrations are similar in stressed animals and controls. We suspect that the normalized glucose- and glycogen levels seen at three weeks post-CSD are mediated by the lowered GP activities, caused by the reduction in glucagon.

## Electronic supplementary material

Below is the link to the electronic supplementary material.

Supplementary Material 1

Supplementary Material 2

## Data Availability

Data is provided within the manuscript or supplementary information files.
