# Supplementary material for: Adaptations in hepatic glucose metabolism after chronic social defeat stress in mice
Source: Sci Rep. 2024 Oct 26;14:25511. doi: 10.1038/s41598-024-76310-3 (PMC11513145; doi:10.1038/s41598-024-76310-3)

**Supplementary Information**

Adaptations in hepatic glucose metabolism after chronic social defeat stress in mice

Meijboom FS, Hasch A, Ruiz de Azua I, Takeno Cologna C, Loopmans S, Lutz B, Müller MB, Ghesquière B, van der Kooij MA.

**Supplementary Table 1.** Hepatic gene expression for PEPCK (phosphoenolpyruvate carboxikinase), GDE (glycogen debranching enzyme), PC (pyruvate carboxylase) and ALT (alanine transferase) in the liver at short- (1 wk post-CSD) and long-term (3 wks post-CSD). N= 7-8/group for the short-term and n= 8-10 for the long-term cohort.

|  | Short-term (1wk post-CSD) | | | Long-term (3 wks post-CSD) | | |
| --- | --- | --- | --- | --- | --- | --- |
| Genes | CTRL | CSD | p | CTRL | CSD | p |
| *PEPCK* | 1.23 ± 0.29 | 1.31 ± 0.29 | 0.85 | 1.09 ± 0.15 | 1.62 ± 0.06 | <0.01 |
| *GDE* | 1.07 ± 0.16 | 0.96 ± 0.10 | 0.57 | 1.05 ± 0.12 | 1.17 ± 0.06 | 0.38 |
| *PC* | 1.14 ± 0.22 | 1.30 ± 0.24 | 0.64 | 1.72 ± 0.48 | 4.81 ± 0.22 | <0.001 |
| *ALT* | 1.10 ± 0.17 | 1.01 ± 0.12 | 0.66 | 1.09 ± 0.15 | 1.62 ± 0.06 | <0.01 |

**Supplementary Figure 1. ^13^C_6_-glucose tracing (%) in the brain for metabolites involved in glycolysis/gluconeogenesis.** Relative ^13^C_6_-labeling in the brain (%) was not affected by CSD for any of the measured metabolites involved in glycolysis/gluconeogenesis including (**a**) G6P (t= 0.96, df= 24, *P*= 0.35), (**b**) F1,6BP (t= 0.85, df= 24, *P*= 0.40), (**c**) DHAP (t= 0.69, df= 24, *P*= 0.50), (**d**) 3PG (t= 0.50, df= 24, *P*= 0.62), (**e**) PEP (t= 2, df= 24, *P*= 0.06), (**f**) pyruvate (t= 0.12, df= 24, *P*= 0.90) and (**g**) lactate (t = 1.17, df= 24, *P=* 0.25). Data are presented as individual values and as mean + SEM (n= 13/group). Student’s t-test for **a-g**. G6P: glucose-6-phosphate; F1,6BP: fructose 1,6 biphosphate; DHAP: dihydroxyacetone phosphate; 3PG: 3-phosphoglyceric acid, PEP: phosphoenolpyruvate.

**
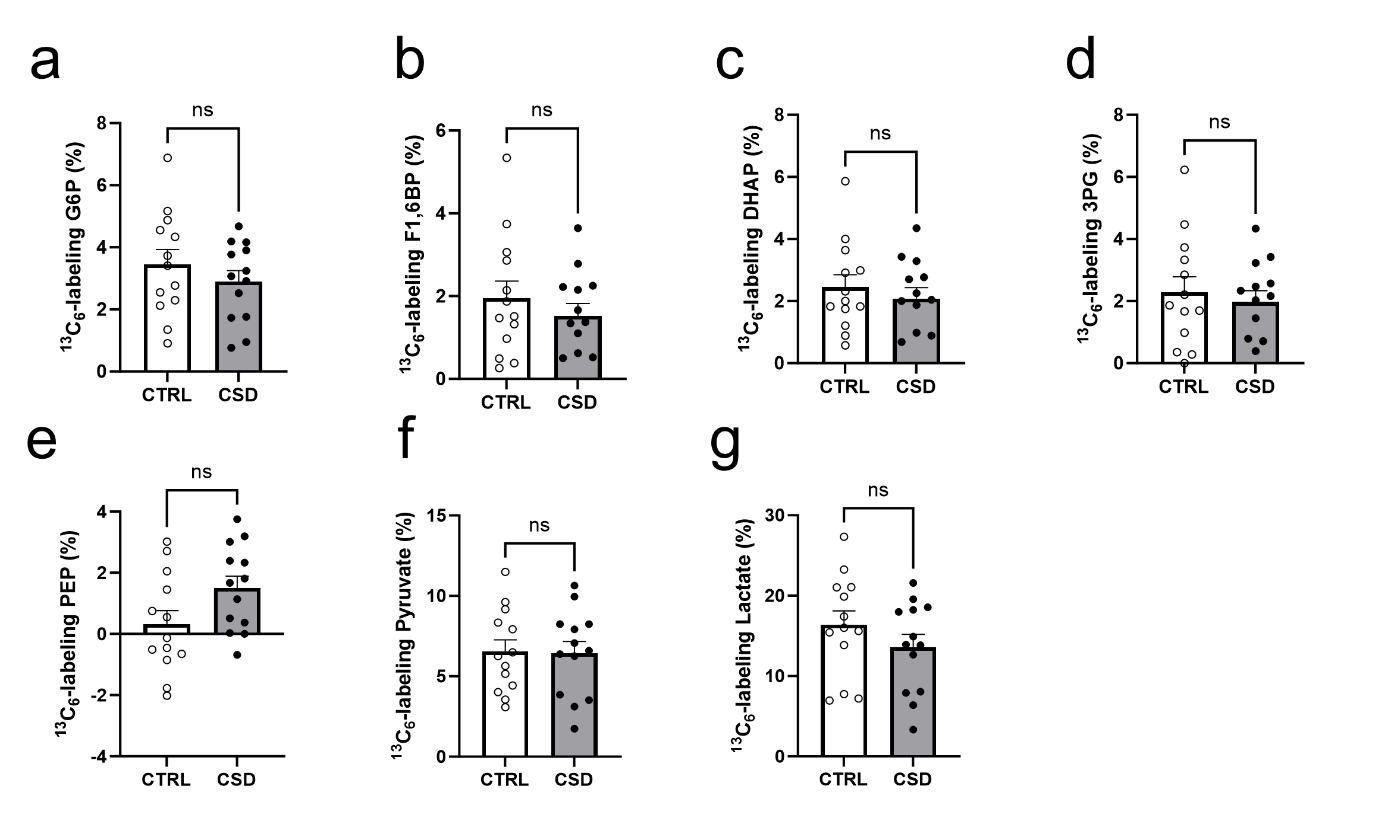
Supplementary Figure 2. ^13^C_6_-glucose tracing (Z-scores) in TCA cycle metabolites in brain and liver.** ^13^C_6_-labeling is expressed as a Z-score (number of standard deviations difference from CTRL), to enhance visibility. Z-scores ± 1.96 (blue shaded area) represent significant differences from CTRL. (**A**) ^13^C_6_-labeling was not altered in striatal brain tissue from stressed mice (Z-scores for ^13^C_6_-labeling in citrate: -0.53, *P*= 0.60; aconitate: -0.50, *P*= 0.61; α-ketoglutarate: -0.55, *P*= 0.58; succinate: -0.15, *P*= 0.88; fumarate: -0.50, *P*= 0.62; malate: -0.47, *P*= 0.64, n= 15/group). (**B**) ^13^C_6_-labeling also did not differ for the liver (Z-scores for ^13^C_6_-labeling in citrate: -0.52, *P*= 0.61; α-ketoglutarate: -0.60, *P*= 0.55; succinate: -0.41, *P*= 0.68; fumarate: -0.43, *P*= 0.66; malate: -0.40, *P*= 0.69, n= 15/group). Data are presented as Z-scores and as mean + SEM.


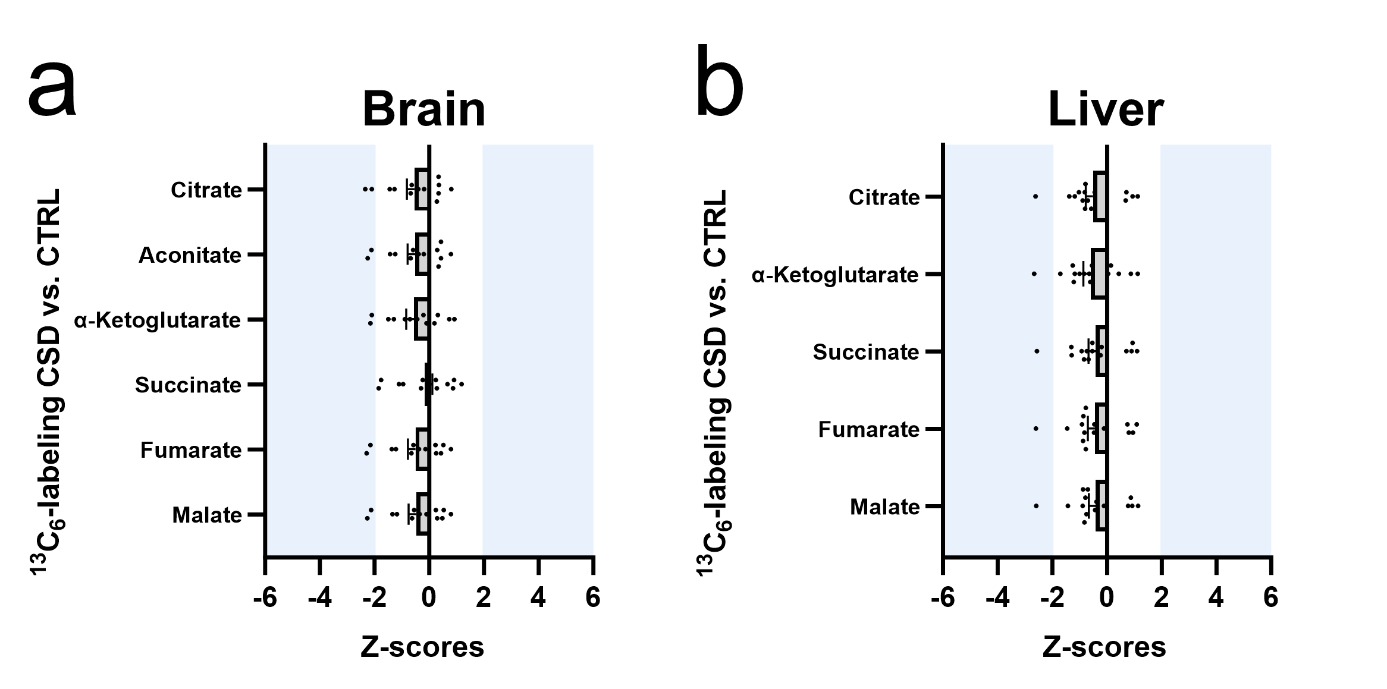


**Supplementary Figure 3. ^13^C_6_-glucose tracing (%) in the brain for metabolites involved in the TCA cycle.** ^13^C_6_-labeling in striatal brain tissue was not affected by stress for metabolites involved in the TCA cycle including (**a**) citrate (t= 1.33, df= 24, *P*= 0.20), (**b**) aconitate (t= 1.26, df= 24, *P*= 0.22), (**c**) α-ketoglutarate (t= 1.34, df= 24, *P*= 0.19), (**d**) succinate (t= 0.39, df= 24, *P*= 0.70), (**e**) fumarate (t= 1.26, df= 24, *P*= 0.22) and (**f**) malate (t= 1.21, df= 24, *P*= 0.24), n= 13/group). Data are presented as individual values and as mean + SEM. Student’s t-test for **a-f**.

**
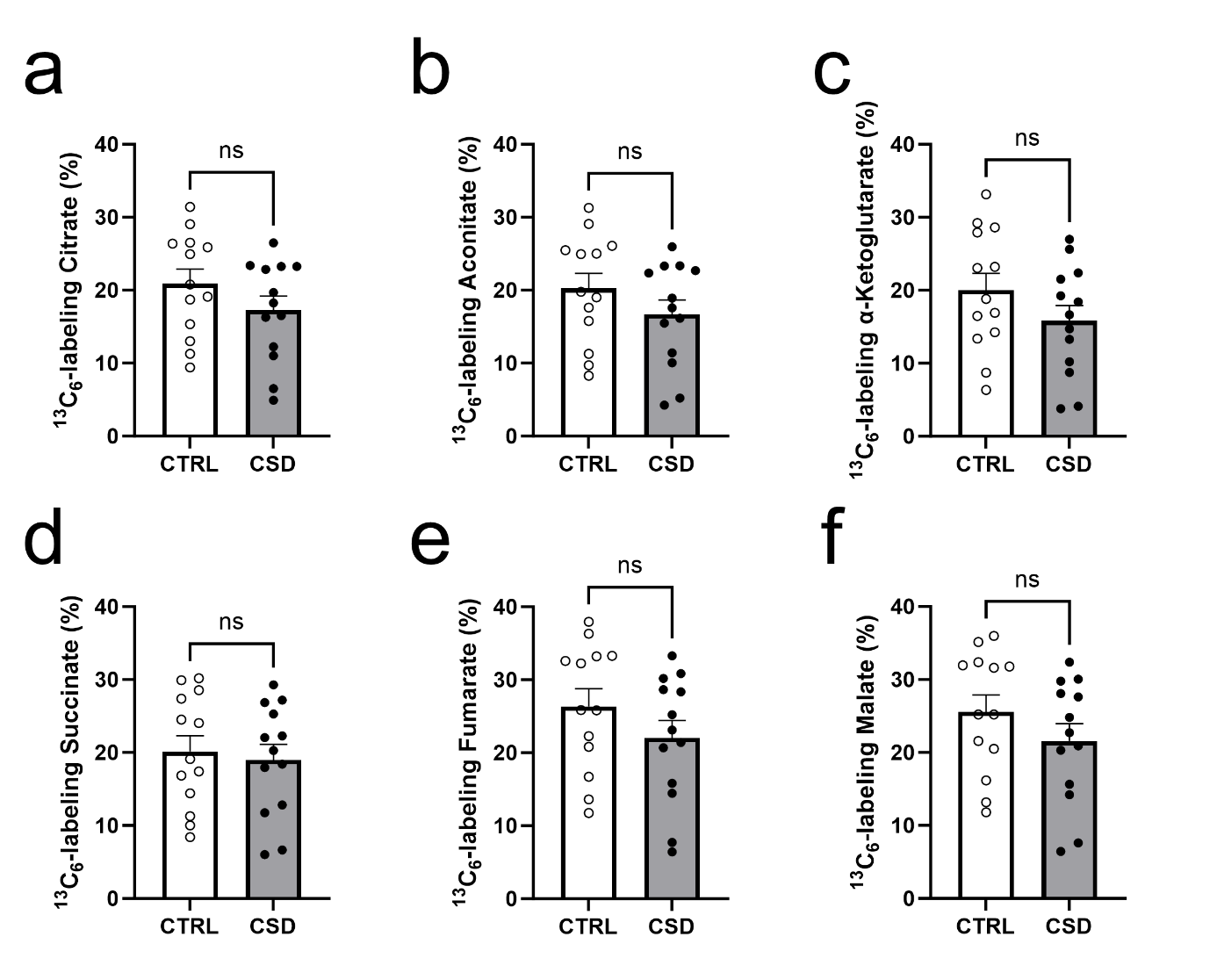
**

**Supplementary Figure 4. ^13^C_6_-glucose tracing (%) in the liver for metabolites involved in the TCA cycle.** ^13^C_6_-labeling in liver tissue was not affected by stress for metabolites involved in the TCA cycle including (**a**) citrate (t= 1.26, df= 27, *P*= 0.22), (**b**) α-ketoglutarate (t= 0.96, df= 27, *P*= 0.34), (**c**) succinate (t= 1.02, df= 27, *P*= 0.32), (**d**) fumarate (t= 1.09, df= 27, *P*= 0.28) and (**e**) malate (t= 1.02, df= 27, *P*= 0.32), n= 14 for CTRL and n= 15 for CSD). Data are presented as individual values and as mean + SEM. Student’s t-test for **a-e**.


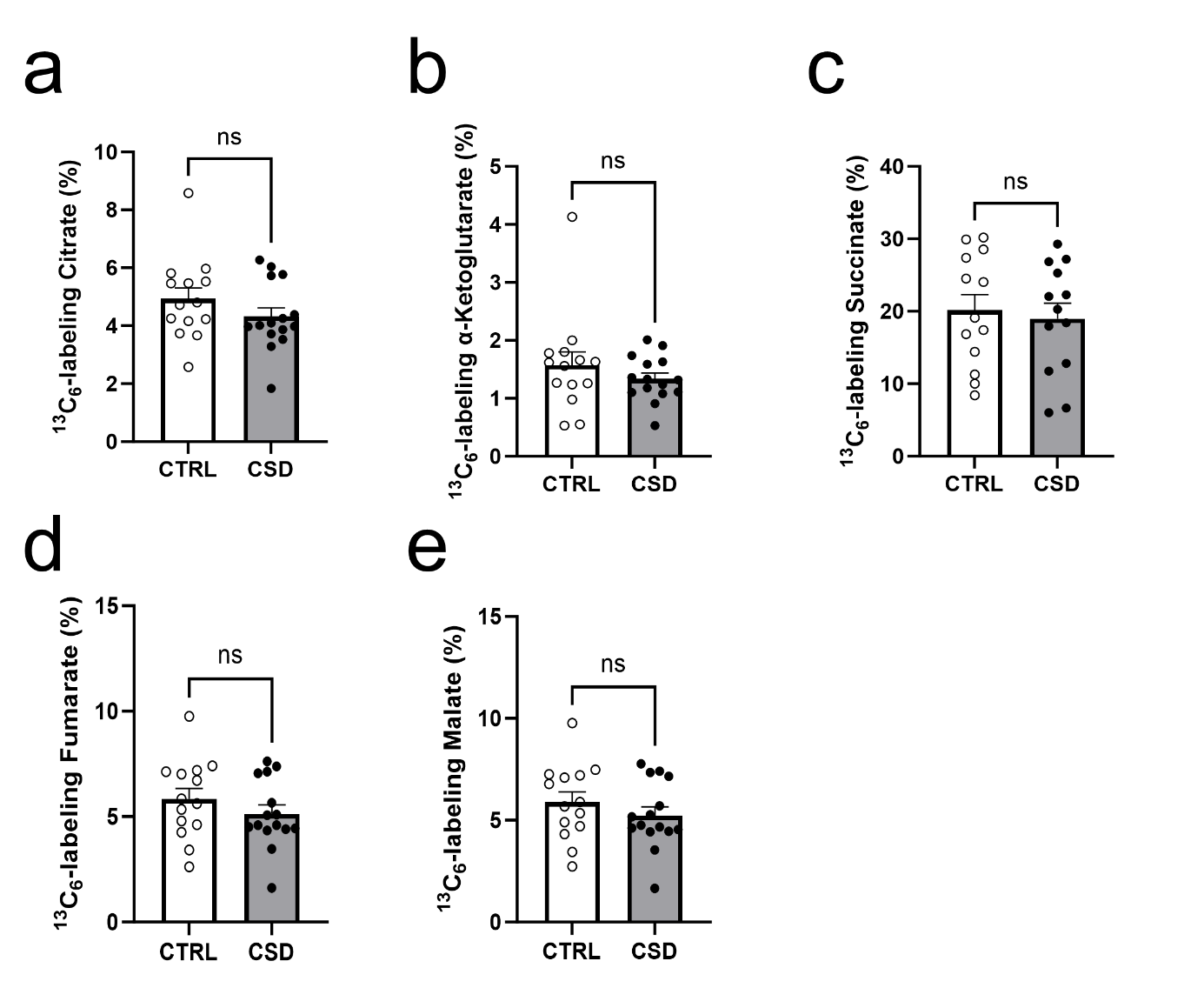


**Supplementary Figure 5. Ambulatory activity displayed in metabolic cages and bodyweight.** (**a**) The distance travelled did not differ between CTRL and CSD-treated mice, one week after stress exposure (F_1,10_= 3.38, *P*= 0.10, n= 6/group). The nocturnal phase is indicated by the grey blocks. (**b**) We did not observe differences in bodyweight between CTRL and CSD-exposed mice before- or after exposure to the metabolic cages (effect of stress: F_1,10_= 0.15, *P*= 0.70, n= 6/group). Two-way ANOVA with repeated measures for **a-b**. Data are presented as mean + SEM.


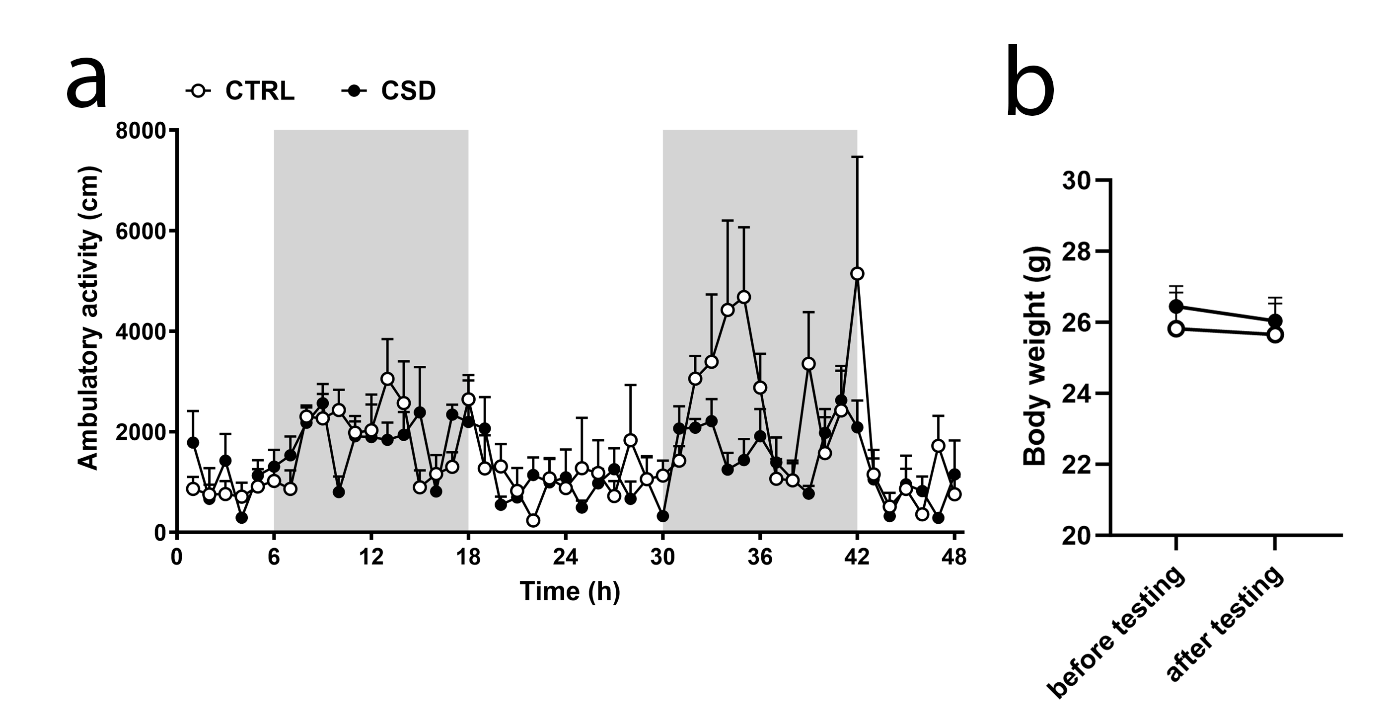

Supplement: Supplementary file 1 — Supplementary Material 1 [file 41598_2024_76310_MOESM1_ESM.docx]
